# Cancer risk among elderly persons with end-stage renal disease: a population-based case–control study

**DOI:** 10.1186/1471-2369-13-65

**Published:** 2012-07-26

**Authors:** Fatma M Shebl, Joan L Warren, Paul W Eggers, Eric A Engels

**Affiliations:** 1 Division of Cancer Epidemiology and Genetics, National Cancer Institute, National Institutes of Health, Department of Health and Human Services, Rockville, MD, USA; 2 Division of Cancer Control and Population Sciences, National Cancer Institute, National Institutes of Health, Department of Health and Human Services, Rockville, MD, USA; 3 Kidney and Urology Epidemiology Program, National Institute of Diabetes and Digestive and Kidney Diseases, National Institutes of Health, Department of Health and Human Services, Bethesda, MD, USA; 4 Department of Chronic Disease Epidemiology, Yale School of Public Health, New Haven, CT, USA

**Keywords:** End-Stage Renal Disease, ESRD, ESKD, Dialysis, Cancer

## Abstract

**Background:**

Patients with end-stage renal disease (ESRD) have elevated cancer risk. Cancer risk increases with age, but associations of ESRD with specific malignancies are incompletely studied for older individuals.

**Methods:**

We conducted a population-based case–control study (1,029,695 cancer and 99,610 controls) among the U.S. elderly using SEER-Medicare linked data. We defined ESRD as presence of dialysis claims in the 3 months prior to selection.

**Results:**

Although ESRD was not associated with excess cancer risk overall (odds ratio 1.02; 95%CI 0.91-1.14), risk was specifically increased for cancers of the stomach (1.45; 1.16-1.81), small intestine (1.92; 1.27-2.92), colon (1.17; 1.00-1.36), liver (1.53; 1.16-2.01), biliary tract (1.78; 1.20-2.65), lung (1.17; 1.02-1.34), cervix (2.12; 1.39-3.23), kidney (2.42; 2.01-2.92), and for multiple myeloma (1.77; 1.40-2.24) and chronic myeloid leukemia (1.74; 1.08-2.80). The association between liver cancer and ESRD was attenuated upon adjustment for hepatitis B and C infection or diabetes mellitus. Multiple myeloma risk was highest with short ESRD duration (p < 0.0001), possibly reflecting reverse causality, while kidney cancer risk showed a borderline rise over time (p = 0.08).

**Conclusions:**

Among elderly individuals with ESRD, the excess risks for some cancers may reflect immune dysfunction or a high prevalence of other risk factors, such as viral infections or diabetes mellitus. Our results underscore the need for studying biological pathways of carcinogenesis in ESRD.

## Background

In 2009, a total of 571,414 people within the U.S. were living with end-stage renal disease (ESRD) [1]. ESRD incidence increases with age, and therefore the prevalence of ESRD is growing most quickly among individuals aged sixty five and older, such that 36% of individuals living with ESRD in the U.S. in 2009 were at least 65 years old [1]. ESRD patients are at high risk of hospitalization and death due to infection, especially related to vascular access, septicemia, and pneumonia; uremia; and cardiovascular disease, which might be attributed to immune system abnormalities in ESRD patients [2,3].

In addition, ESRD patients seem to a have modestly increased risk of cancer, with overall standardized incidence ratios (SIRs, which measure risk relative to the general population) ranging from 0.9 to 1.5 [4-10]. Malignancies for which risk may be more substantially increased include cancers of the kidney, bladder, and thyroid; multiple myeloma; and virus-related malignancies, specifically Kaposi sarcoma, non-Hodgkin lymphoma, and Hodgkin lymphoma. A large multi-national study used U.S. data from the United States Renal Disease System (USRDS) and incorporated Medicare claims records to ascertain the occurrence of cancer [5]. This approach was limited by potential inaccuracies in Medicare claims for cancer diagnoses, leading to possible confusion between incident and prevalent cancers [11]. The study reported an elevated risk for cancer among individuals with ESRD and documented attenuation with advancing age in the magnitude of this association. However, the authors did not present results for individual cancer types separately for people who were age 65 years or older [5].

The potential association of ESRD and cancer risk is particularly important for the elderly, because with the aging of the U.S. population, there is an increase in the number and proportion of older adults living with ESRD, and also because the incidence of most cancers increases with age. However, because the epidemiology of cancer among elderly ESRD patients has not been evaluated in detail, it is unclear whether ESRD increases cancer risk in this population, and if so, for which cancers the increase is present. We therefore conducted the present study, using linked data from cancer registries and Medicare, to assess cancer risk related to ESRD among elderly individuals in the U.S.

## Methods

### SEER-Medicare dataset

This study evaluates cancer risk in elderly ESRD patients living in Surveillance, Epidemiology, and End Results (SEER) cancer registry areas. The National Cancer Institute oversees and supports the SEER program (http://seer.cancer.gov/) which is a consortium of U.S. population-based cancer registries. SEER originated in 1973 and over time expanded registry coverage to nearly 26% of the U.S. population. Medicare is a federally sponsored insurance program that provides primary health insurance for approximately 97% of the U.S. population aged 65 years and older. In addition, Medicare covers individuals with ESRD or disability regardless of their age (http://www.cms.hhs.gov/).

Details of the SEER-Medicare dataset (http://healthservices.cancer.gov/seermedicare/) have been published elsewhere [11]. Briefly, the SEER-Medicare database links data on SEER cancer cases with Medicare to retrieve claims data on Medicare-covered individuals. The SEER registry data contain demographic and clinical information on all incident cancer cases. Medicare data include claims from 1986 forward for inpatient hospitalizations (Part A), and 1991 forward for physician and outpatient services (Part B). These claims use International Classification of Diseases (ICD-9), current procedures terminology (CPT-4) codes, and revenue center codes to bill for services and related diagnoses. Comparable Medicare data are also available for a cohort of cancer-free individuals comprising a 5% random sample of Medicare beneficiaries residing in SEER areas.

### Study design and subject selection

We used the SEER-Medicare data to conduct a population-based case–control study of risk of cancer among the elderly. As described in detail elsewhere [12], this study design is equivalent to a cohort study of Medicare recipients, from which we selected all cancer cases and a random sample of cancer-free individuals, and it has the advantage of computational efficiency. Thus, we identified all cancer cases from individuals diagnosed in SEER with a first malignancy during 1992–2005 who were aged 66–99 years at diagnosis. Cases were required to have at least 12 months of Medicare data prior to diagnosis (1 year of prior Part A, Part B Medicare coverage while not enrolled in a health maintenance organization [HMO]) so that there was sufficient time in which to identify ESRD information prior to the cancer diagnosis. We excluded individuals whose cancer was diagnosed only on autopsy or death certificate, since these cases might be incidental or have atypical behavior. We excluded cancer cases from the analysis if they had a prior Medicare claim indicating a history of organ transplant or were ever infected with human immunodeficiency virus, to assure that cases were not due to known causes of immunosuppression. Cancers were classified using International Classification of Diseases for Oncology (third edition, ICD-O3) topography and morphology codes and grouped using the SEER “site recode with Kaposi sarcoma and mesothelioma.”

We randomly selected 100,000 controls from the 5% sample of Medicare beneficiaries living in SEER areas to match the total group of cancer cases. Specifically, controls were required, as of July 1 in the calendar year of selection, to be alive, free of any SEER-reported malignancies, and have at least 12 months of prior non-HMO, Part A, Part B Medicare coverage. From this eligible group, we then selected controls who were frequency matched to the entire group of cancer cases by individual calendar year of diagnosis, gender, and age in five categories (66–69, 70–74, 75–79, 80–84, 85–99 years). A subject could be selected as a control multiple times for cases in different calendar years and could later become a cancer case. Following selection of 100,000 controls, we excluded individuals if they had a prior Medicare claim of organ transplant or were ever infected with human immunodeficiency virus.

### Ascertainment of ESRD and other medical conditions

For each case and control subject, we defined ESRD to be present based on Medicare claims for chronic dialysis care that was consistent with ESRD. We used this ascertainment tactic because there were no ICD-9 diagnosis codes in the Medicare claims files specific to ESRD. Specifically, we required that a claim from a physician or hospital outpatient facility include one of the following codes: CPT-4 code for dialysis of 90925 (ESRD service for full month), 90935–90937 (hemodialysis service with physician evaluation), 90945–90947 (dialysis [other than hemodialysis] service with physician evaluation), 90966 (services for home dialysis), or 90997–90999 (hemoperfusion, unlisted dialysis procedure); or revenue center codes 0821–0825, 0829–0835, 0839–0845, 0849–0855, or 0859. We required a minimum of one ESRD or dialysis claim in each of the three months immediately prior to cancer diagnosis or control selection. Our list of codes is similar to those used previously by Taneja et al. to study the distribution of cancer stages among ESRD patients [13], but it is more restrictive, because Taneja et al. required the presence of codes for both chronic renal disease and dialysis, whereas we did not require both types of codes but required repeated claims over three months.

For each individual, we searched Medicare claims files for other conditions related to ESRD or specific cancers. These conditions included obesity (ICD-9: 278.0x), diabetes mellitus (250.0x), hypertension (401.x-405.x), peptic ulcer (531.x-534.x), alcoholic liver disease (571.0-571.3), hepatitis C virus infection (HCV, 070.41, 070.44, 070.51, 070.54, 070.70, 070.71, V02.62), hepatitis B virus infection (HBV, 070.2, 070.3), hemochromatosis (275.0), cholelithiasis (574.x), pernicious anemia (281.0, 281.1), regional enteritis (555.x), ulcerative colitis (556.x), diverticular disease (562.x), and various kidney diseases (753.x, 580.x-584.x, 587). Conditions were determined to be present based on Medicare claims in inpatient, outpatient, or physician files. We excluded the 3-year period prior to cancer diagnosis/selection to focus on conditions that were longstanding and therefore most likely relevant to modify the association between ESRD and cancer risk.

### Statistical analysis

To compare ESRD prevalence in cancer cases and controls, we utilized unconditional logistic regression models to obtain odds ratios (ORs) and 95% confidence intervals (CIs). We fitted separate logistic models for each cancer type and used all controls in each analysis. For cancers that were found to be associated with ESRD, we assessed cancer risk according to the apparent duration of ESRD based on the date of the earliest supporting Medicare claim. We also evaluated models that adjusted for the presence of other medical conditions related to ESRD or the cancers of interest.

All logistic models were adjusted for age, gender, calendar year of cancer diagnosis/control selection, and race. In addition, we adjusted the OR variance to accommodate the repeated selection of individuals as controls, and the selection of some controls who later served as cases [12]. To avoid sparse data and because the SEER-Medicare data use agreement prevents presenting results for 10 subjects or fewer, we did not analyze data for cancers with fewer than 3000 cases (based on an ESRD prevalence of 0.3% in controls, this number corresponds to an expected 10 cancer cases with ESRD under the null hypothesis of no association). For prostate cancer, we also show associations with ESRD stratified by stage of cancer diagnosis and according to whether a prior Medicare claim for prostate-specific antigen (PSA) testing was present.

## Results

The study included 1,029,732 cancer cases and 99,615 controls (Table 1). Cases and controls were well-matched on age, gender, race/ethnicity, and calendar year. The majority of subjects were non-Hispanic whites (cases 86%, controls 84%), with a median age of 76 years in cases and 75 years in controls.

**Table 1:** Characteristics of cancer cases and controls

	**Cancer cases**	**Controls**
	**(n = 1,029,732)**	**(n = 99,615)**
Age in years, n (%)		
66-74	452,889 (44.0%)	47,389 (47.6%)
75-84	434,024 (42.2%)	39,378 (39.5%)
85-99	142,819 (13.9%)	12,848 (12.9%)
Median age	76	75
Gender, n (%)		
Male	544,023 (52.8%)	54,232 (54.4%)
Female	485,709 (47.2%)	45,383 (45.6%)
Selection year, n (%)		
1992-1994	169,248 (16.4%)	14,893 (15.0%)
1995-1998	198,879 (19.3%)	19,775 (19.9%)
1999-2005	661,605 (64.3%)	64,947 (65.2%)
Race/ethnicity, n (%)		
White	885,788 (86.0%)	83,730 (84.1%)
Black	80,226 (7.8%)	6,924 (7.0%)
Asian	24,980 (2.4%)	3,844 (3.9%)
Hispanic	14,992 (1.5%)	2,457 (2.5%)
Native American Indian	2,398 (0.2%)	321 (0.3%)
Other/unknown	21,345 (2.1%)	2,339 (2.4%)

ESRD was reported in 3,582 (0.35%) cancer cases and 357 (0.36%) controls. ESRD was thus not associated with an overall increase risk of cancer (OR 1.02, 95% CI 0.91-1.14; Table 2). Based on the earliest Medicare claim for ESRD or dialysis, the median duration of ESRD was 25 months (interquartile range 12–45) in cases and 25 months (interquartile range 14–43) in controls.

**Table 2:** Associations between ESRD and cancer risk

	**N**	**ESRD**		**OR (95%CI)**	**P value**
		**N**	**%**		
**Controls**	99,615	357	0.36	ref	--
**Overall cancer**	1,029,732	3582	0.35	1.02 (0.91, 1.14)	0.8
**Specific cancer site:**					
Tongue	4,007	18	0.45	1.32 (0.82, 2.13)	0.3
Gum and other mouth	3,407	<11	<0.4	0.95 (0.50, 1.78)	0.9
Esophagus	10,261	39	0.38	1.02 (0.73, 1.43)	0.9
Stomach	20,106	118	0.59	1.45 (1.16, 1.81)	0.001*
Small intestine	3,502	24	0.69	1.92 (1.27, 2.92)	0.002
Colon	104,002	390	0.37	1.17 (1.00, 1.36)	0.05
Rectum	33,086	91	0.28	0.82 (0.65, 1.04)	0.11
Liver	9,034	63	0.70	1.53 (1.16, 2.01)	0.003
Gallbladder	3,430	15	0.44	1.32 (0.78, 2.22)	0.3
Other biliary	4,389	27	0.62	1.78 (1.20, 2.65)	0.004
Pancreas	29,765	122	0.41	1.21 (0.98, 1.50)	0.07
Larynx	7,431	33	0.44	1.15 (0.80, 1.66)	0.4
Lung	161,687	661	0.41	1.17 (1.02, 1.34)	0.02
Soft tissue (including heart)	4,202	<11	<0.4	0.56 (0.28, 1.12)	0.1
Melanoma of skin	24,626	75	0.30	1.07 (0.82, 1.41)	0.6
Other non-epithelial skin	3,585	17	0.47	1.58 (0.96, 2.59)	0.07
Breast	122,315	339	0.28	1.11 (0.90, 1.38)	0.3
Cervix	3,686	28	0.76	2.12 (1.39, 3.23)	<0.001*
Uterine corpus	24,507	66	0.27	1.05 (0.77, 1.43)	0.8
Ovary	14,683	40	0.27	1.07 (0.75, 1.54)	0.7
Prostate	193,632	336	0.17	0.42 (0.35, 0.50)	<0.001*
Bladder	55,559	218	0.39	1.20 (1.00, 1.44)	0.05
Kidney and renal pelvis	22,999	185	0.80	2.31 (1.92, 2.78)	<0.001*
Kidney	20,684	177	0.86	2.42 (2.01, 2.92)	<0.001*
Renal pelvis	2,315	<11	<0.4	1.13 (0.56, 2.28)	0.7
Thyroid	5,520	25	0.45	1.29 (0.85, 1.93)	0.2
Non-Hodgkin lymphoma	50,513	120	0.24	0.77 (0.62, 0.96)	0.02
Multiple myeloma	13,949	92	0.66	1.77 (1.40, 2.24)	<0.001*
Acute myeloid leukemia	7,571	25	0.33	1.02 (0.67, 1.53)	0.9
Chronic myeloid leukemia	3,271	18	0.55	1.74 (1.08, 2.80)	0.02

Abbreviations: ESRD end-stage renal disease, OR odds ratio, CI confidence interval.

Odds ratios are adjusted for age, race, sex and selection year. Cancers with less than 3000 cases are not shown.

* P-value is significant at Bonferroni p-value threshold of 0.002.

Nonetheless, with respect to specific cancers (Table 2), ESRD was associated with significantly elevated risk for cancers of the stomach (OR 1.45), small intestine (1.92), colon (1.17), liver (1.53), biliary tract (1.78), lung (1.17), and cervix (2.12), as well as for multiple myeloma (1.77) and chronic myeloid leukemia (1.74). As shown in Table 2, elevated risk was present for cancers of the kidney and renal pelvis (OR 2.31), but this increase was limited to cancers of the kidney itself (OR 2.42) and was not significant for the renal pelvis (OR 1.13). Among the kidney cancers, 18,707 (90.4%) were renal cell carcinomas, 633 (3.1%) were transitional cell carcinomas, and 1,344 (6.5%) were other/unspecified subtypes. Among renal pelvis cancers, 2,164 (93.5%) were transitional cell carcinomas, and 115 (5.0%) were other/unspecified subtypes. Analysis of these specific histologic subtypes of kidney and renal pelvis cancers revealed an excess risk for renal cell carcinoma in association with ESRD (N = 18,743 cases, OR 2.38, 95% CI 1.96-2.89), but not for transitional cell carcinoma (N = 2,797 cases, OR 0.93, 95% CI 0.46-1.87). In contrast, ESRD was associated with reduced risk for prostate cancer (OR 0.42) and non-Hodgkin lymphoma (OR 0.77) (Table 2).

Of all these cancers, only multiple myeloma showed a significant change in risk with duration of ESRD. For multiple myeloma, there was a substantial decline with prolonged ESRD, such that risk was highest immediately after the first Medicare claim for dialysis, with ORs of 3.74 in the 0–12 months, 1.89 in the 12–25 months, 0.89 in the 25–44 months, and 0.79 in the 44+ months after first claim (p < 0.001). There was a borderline trend of increasing risk of kidney cancer with longer duration of ESRD (ORs 1.85 in the 0–12 months, 1.75 in the12-25 months, 1.61 in the 25–44 months, and 3.57 in the 44+ months after first claim, p = 0.08).

We evaluated whether associations with ESRD were affected when we adjusted for the presence of other medical conditions. Adjustment for the presence of obesity did not change the observed associations with ESRD for any cancers (data not shown). Likewise, associations with ESRD were not materially affected when we made the following model-based adjustments (data not shown): risks of stomach and esophagus cancers when adjusted for history of peptic ulcer or pernicious anemia; risk of colon cancer when adjusted for diverticular disease, ulcerative colitis or regional enteritis; risks of renal and urinary bladder cancers when adjusted for history of kidney disease or hypertension; and risk of biliary tract cancer upon adjusting for the presence of gallstones.

For liver cancer, the association with ESRD remained significant after adjustment for alcoholic liver disease or hemochromatosis (data not shown). However, the association between ESRD and liver cancer was attenuated after adjustment for HBV, HCV infection, or diabetes mellitus (adjusted ORs 1.25, 1.13, and 1.15 respectively).

We also examined associations with ESRD for prostate cancer according to cancer stage. ESRD patients manifested a reduced risk of localized/regional cancer (OR 0.34), but not risk of distant/metastatic (OR 0.89) or unstaged cancer (OR 0.84) (Table 3) Among controls, men with ESRD were less likely than men without ESRD to have a claim for a PSA test in the one-year period prior to selection (24.1% vs. 35.5%, OR 0.58, 95% CI 0.43-0.78). Among prostate cancer cases, a claim for PSA testing was present in the year prior to diagnosis in 59.2% and 67.9% (OR 0.69, 95% CI 0.55-0.85) of those with and without ESRD, respectively. Risk of prostate cancer (in particular, localized/regional cancer) was significantly lower among individuals with ESRD compared to controls regardless of their screening status (Table 3).

**Table 3:** Associations between ESRD and prostate cancer, by stage at cancer diagnosis and PSA screening

**Prostate cancer outcome**	**Number of cases**	**Association with ESRD, odds ratio (95% CI)**		
		**Overall**	**PSA in 1 year before diagnosis/selection**	**No PSA in 1 year before diagnosis/selection**
All cancers	193,632	0.42 (0.35, 0.50)	0.52 (0.39, 0.71)	0.52 (0.42, 0.66)
By stage				
Localized/regional	136,904	0.34 (0.28, 0.41)	0.45 (0.33, 0.62)	0.42 (0.31, 0.55)
Distant/metastic	8,333	0.89 (0.62, 1.26)	0.98 (0.54, 1.79)	0.95 (0.61, 1.48)
Unstaged	8,302	0.84 (0.58, 1.22)	1.12 (0.66, 1.89)	0.91 (0.50, 1.66)

Abbreviations: ESRD end-stage renal disease, CI confidence interval, PSA prostate specific antigen test.

## Discussion

In this population-based case–control study among elderly adults, we found that ESRD was not associated with an overall increased risk of cancer. These results differ from those seen in most previous studies [5,6,14], which have shown a modest overall increased risk of cancer. This difference may be explained by the restriction of our study to elderly adults. As noted above, Maisonneuve et al. reported an attenuation with age in the association between ESRD and cancer risk, such that in the U.S. the overall SIR for cancer declined from 4.6 for those whose age at first dialysis was <35 years to 1.1 for individuals who were age 65 years or older [5]. This overall attenuation could be due to the increase of background cancer rates with age, but the results likely differ for individual cancer types.

Indeed, although overall cancer risk was not elevated in association with ESRD, we did see increased risks for certain cancers such as cancers of stomach, small intestine, colon, liver, biliary tract, lung, cervix, and kidney, as well as for multiple myeloma and chronic myeloid leukemia, where risks were elevated 1.16 to 2.40-fold. Among cancers of the kidney and pelvis, we found an approximately 3-fold excess of renal cell carcinoma but no increase for transitional cell carcinoma. Previous studies reported an excess risk of several of these malignancies, including multiple myeloma and bladder and kidney cancers [4-6,15,16]. Some prior studies found increased risks for other cancer that we did not find associated with ESRD, including thyroid cancer, non-Hodgkin lymphoma (for which we found a modest inverse association), Hodgkin lymphoma, and Kaposi sarcoma [5,14]. Again, the differing magnitudes of association between prior studies and our study could potentially be attributed to our subjects’ advanced age, while other studies included a wider age range.

Notably, the increased risks that we observed for several infection-related cancers (i.e., liver, stomach, and cervical cancers) could reflect immunodeficiency associated with ESRD. Chronic uremia leads to metabolic abnormalities that alter the immune response such that antigen-presenting cell (APC) function is impaired, lymphocyte survival is shortened, proliferation of T-cells is impaired, together with an increased suppressor T-cell activity, decreased helper T-cell (Th) activity, and increased Th1/Th2 ratio [2,3]. Nutritional abnormalities that are prevalent among individuals with ESRD, such as selenium deficiency, reduced glutathione peroxidase activity, and vitamin D deficiency [17-20] could also play a role in development of cancer, particularly for colon cancer [21-23]. Individuals with ESRD commonly receive erythropoietin for treatment of anemia, which might be implicated in carcinogenesis and could potentially explain some of the observed excess cancer risk among individuals with ESRD [24].

However, arguing against a biological effect underlying some of these associations, we did not observe an increase in cancer risk with longer duration of ESRD. Another possibility is that, rather than reflecting the effects of ESRD itself, the high risk of some cancers may arise from a high prevalence of established cancer risk factors (e.g., infections with human papillomavirus [cervical cancer], HBV and HCV [liver cancer], or tobacco use [lung cancer]) [25-28]. Indeed, this possibility is supported by our observation that the association between ESRD and liver cancer was attenuated after we adjusted for HCV infection, HBV infection, or diabetes mellitus.

In addition, excess risks were observed for malignancies (i.e., bladder and kidney cancers, multiple myeloma) that could directly lead to ESRD or are related to such conditions. Cancers of the renal pelvis and bladder may be attributed to excess analgesic consumption, a cause of renal failure [9,29,30]. Likewise, polycystic kidney disease, a cause of renal failure, predisposes to renal cell carcinoma [31,32]. Nonetheless, ESRD itself probably causes some kidney cancers, as the development of multiple renal cysts during dialysis, with the subsequent occurrence of renal cell carcinoma, is well-described [33-40]. This notion is further supported by the study of Maisonneuve et al. [5] that detected an increased risk of kidney cancer with increasing duration of dialysis.

In contrast, the strong increase in multiple myeloma risk in the first 1–2 years after ESRD diagnosis, and declining risk over longer time intervals, could be attributed to reverse causality, such that multiple myeloma leads to renal failure. This situation could arise if multiple myeloma was undiagnosed at initiation of dialysis, or if the diagnosis date is in error in the cancer registry due to delayed reporting. Maisonneuve et al. also noted the excess of multiple myeloma cases and likewise attributed this observation to prevalent cases [5]. Finally, treatment of glomerulonephritis with azathioprine or cyclophosphamide may predispose to certain types of cancers such as kidney cancer, bladder cancer, and multiple myeloma [41,42].

Our findings of markedly low risk of prostate cancer deserve comment. A biologically protective effect of ESRD seems unlikely, because the inverse association was present only for localized cancer and not for distant/metastatic cancer. We believe that the inverse association instead may be a manifestation of screening. Recommendations for prostate cancer screening using PSA testing and digital rectal examination are presently in flux, but screening has typically been considered only for men who have an extended life expectancy. One possibility is that, due to their lower life expectancy, men with ESRD do not receive screening with the PSA test as frequently as other men, which could lead to an apparent deficit of prostate cancer. Indeed, it is estimated that 40% of prostate cancer cases in the U.S. are detected through PSA testing [43]. Among controls, we found a lower prevalence of claims for testing among men with ESRD, which supports that the apparent diminished risk is partly due to lack of screening. However, we also found a lower risk for prostate cancer associated with ESRD among men who received PSA testing, which could potentially be explained by decreased sensitivity of PSA testing in men with ESRD. This notion is supported by Bruun et al. [44], who reported that individuals with chronic kidney disease have elevated levels of free PSA, which is interpreted (when the total PSA is elevated) to indicate the presence of benign prostate disease. In addition, the association between ESRD and reduced risk of prostate cancer among apparently unscreened men may partly reflect confounding due to unmeasured screening that we did not capture. In summary, we believe that the reduced risk of localized/regional prostate cancer in men with ESRD may be explained by less frequent screening or reduced sensitivity of screening. However, we do not advocate for increasing the frequency of screening among men with ESRD. Recently the U.S. Preventive Services Task Force (USPSTF), opposed the use of PSA for prostate cancer screening regardless of age because the harms overweigh the benefits [45]. Given, the overall poor survival of men with ESRD, PSA-based prostate cancer screening will not be of additional value.

Our study has several important strengths, including the ability to assess all type of cancers and its large size. Our study is representative of the elderly U.S. population, since it includes all cancers from SEER cancer registries and a random sample of controls with Medicare from the same geographic areas [12]. The use of SEER allowed comprehensive ascertainment of the occurrence of cancers, since these cancer registries achieve largely complete surveillance and provide high-quality data on incident cancers.

Limitations of our study should also be noted. For instance, SEER does not capture squamous cell and basal cell carcinoma of the skin. In addition, we were unable to assess cancer risk in people younger than 66 years old, so our results are not generalizable to a younger population. Furthermore, because ESRD is somewhat uncommon, we may have missed some associations with cancer. Ascertainment of ESRD in our study was likely complete, because Medicare pays for dialysis care for most U.S. ESRD patients. Our use of Medicare claims is supported by the similar prevalence of ESRD among our controls and reported by the USRDS program for the same age group (0.31% of our controls vs. 0.21-0.44% during 1992–2005) [46]. However, because we used dialysis claims to identify people with ESRD, we may have inadvertently included some people with less severe kidney disease. In addition, we did not have the capability to fully determine duration of ESRD because Medicare claims were only available starting in 1991. Another limitation that should be considered is the possibility that competing mortality from secondary complications of dialysis would attenuate the association between ESRD and cancer. This effect would occur in any study of the ESRD population, and the results should be interpreted to reflect risk of cancer among surviving patients. A final limitation is that we made multiple comparisons, and some associations might be due to chance. Fewer associations would be statistically significant using a conservative p-value threshold of 0.002 (based on a Bonferroni correction for 31 cancer types, see Table 2).

Our results may help inform clinical decisions about cancer screening. Given the lower risk of prostate cancer in men with ESRD, and the lower life expectancy of these men relative to the general population there is no indication that men with ESRD should receive PSA testing. These findings also do not support more aggressive screening for other common cancers, because there was no increased risk for breast cancer, and only a modest increase in colon cancer risk. Similarly, others have suggested that in the circumstances of high morbidity and mortality from other diseases, screening for kidney cervical, colon, and breast cancers is less cost-effective than other health interventions in the ESRD population [47-49].

## Conclusions

In conclusion, we conducted the first comprehensive population-based study of cancer risk associated with ESRD in the elderly. Our results document an elevated risk of some types of malignancy. Additional research on the etiologic roles of disturbed immunity, viral infections, and the biological pathways underlying the development of cancer in ESRD is needed.

## Competing interests

All the authors declare no conflict of interest.

## Authors’ contributions

All authors contributed to the conception and design of the study. FMS analyzed the data. FMS and EAE drafted the manuscript. All authors contributed to the critical review of the manuscript. The final submitted version of the manuscript was approved by all authors.

## Pre-publication history

The pre-publication history for this paper can be accessed here:

http://www.biomedcentral.com/1471-2369/13/65/prepub
